# Impressions in Plaster, Wax, and Gutta Percha

**Published:** 1849-04

**Authors:** 


### IMPRESSIONS IN PLASTER, WAX, AND GUTTA PERCHA.

WE have now these three articles, all of which are used, and recommended by the profession, for taking impressions of the mouth. We design making a few remarks on each. An article, however, in the October number of the American Journal and Library of Dental Science, for 1848, by the Cazenovia editor, describes so well the manner of getting a correct impression with the plaster, as well as the kind and quality to be used, that we shall give his remarks entire, and say but little on the use of this article, merely making one or two suggestions to those who have not, as yet, attempted this operation. In preparing our cup for this operation, we select one as well adapted to the mouth as possible; then, with adhesive wax, close up the end which passes back across the palital arch; the cup is then introduced into the mouth, and pressed to that position we design it to occupy, when getting the impression with the plaster; the wax may be extended entirely around the border of the cup. This retains the plaster, and keeps it from flowing into the mouth. The necessity of first applying a portion of plaster to the roof of the mouth, and after the plaster has set, the introduction of a probe, as directed by the Cazenovia editor, we have found indispensable.

In getting impressions of the lower jaw, we have found, when the depression was great, on one or both sides, from where the molar teeth had been extracted, that, by first applying a portion of plaster to these places, and then the introduction of the cup, with the plaster just sufficiently set not to pour out, by its proper introduction to the mouth, that a perfect impression is more apt to be obtained.

The difficulty in withdrawing the impression from the mouth, where teeth remain, has kept us from using the plaster in such cases; yet, we have determined to obviate this difficulty, the first time we have trouble to get a suction plate, in the use of our wax impressions, in the following manner. Dry the mouth, and particularly the necks of the teeth, which may be in the way of removing a plaster impression, and wrap around these enough adhesive wax to enlarge the necks, so that the plaster may draw from them easily. A correct impression may thus be obtained of that portion of the mouth designed to be covered by the plate. If the plaster impression was properly dried, could not the metal used for the male cast be poured directly into it? The old method of casting was somewhat similar. The pressure, to secure a good impression with plaster, is not, we think, so great, as with wax, or gutta percha, hence less displacement of the softened portion of the gum takes place. Some patients cannot well retain the cup, with plaster, sufficiently long in the mouth, to harden; any substance, indeed, pressed back far enough in the mouth, to get a proper impression, will gag or nauseate them. For such, we always use wax.

But we give the article of Dr. Dwinelle, on the method of taking plaster impressions, as well as the quality and properties of the article, which are well worth the attention of every dentist:

"Impressions in Plaster.—The usual method of taking impressions in plaster is doubtless generally known to most of our readers; but a few further, and perhaps not altogether familiar hints on the subject, may not be unacceptable to those who fail of complete success in this department.

"The article of gypsum, or plaster of paris, differs essentially in its character; first, according to the locality from which it is obtained; and, secondly, in the manner in which it is prepared for use.

"Pure gypsum is found to consist of lime, 28; sulphuric acid, 40; water, 18; its full equivalent being a fraction over 86. This is the article most commonly in use in our country, under the name of plaster of paris; though our market is mostly supplied from Nova Scotia. Pure gypsum is found, however, in various parts of Europe. The Nova Scotia plaster is obtained by grinding the crystals of gypsum (selenite) to a powder in a mill, and then heating it in a kettle, up to a little above 300 deg. Fahr., (it will boil before it reaches this temperature,) when its two equivalents of water of crystallization are expelled, and it is fit for use. When mixed with water to the consistence of cream, it absorbs its two equivalents of water, and becomes recrystallized, though in an opaque form. If heated to a temperature over 400 deg. Fahr., the particles become indurated, and it no longer possesses the property of absorbing water. But this article is comparatively soft and brittle, is easily decomposed by heating, and is incapable of withstanding the influences of the weather.

"The true plaster of paris, or calcareous gypsum, is of course imported from the vicinity of Paris. It contains about 12 per cent. of carbonate of lime. It is prepared by being *baked* and afterwards ground. When in the form of paste, it is exceedingly plastic, and when consolidated, remarkably hard and tough, owing, do doubt, to the presence of the carbonic acid contained in the carbonate of lime. It is used in Paris extensively, as an out-of-door building material, where it is exposed with impunity to all of the influences of the weather. It will also withstand a high temperature of heat without being impaired. All of these peculiarities render it infinitely superior to the Nova Scotia plaster for our purpose of taking impressions.

"The calcareous gypsum, or Paris plaster, is readily distinguished from the Nova Scotia, in that the latter is of a very clear white color, inclining to blue; it has also a feeling of roughness or grittiness, while the Paris plaster is of a decided yellowish white, and has a soapy feeling when passed through the fingers. Its plasticness, when mixed with water, will at once distinguish it from any other kind. We assure our readers that after making a trial of this article for the purposes mentioned, they would have no inclination to use any other. The plaster should always be finely pulverized and sifted, and the mouth wiped dry, previous to taking impressions.

"In taking impressions in plaster, it often happens that the mould obtained is entirely perfect, except those parts corresponding with the highest point of the roof of the mouth; this imperfection is occasioned by air being concentrated in that locality, in consequence of the cup-shape formation of the jaw, given to it by the alveolar ridge. This can easily be avoided, and success always rendered certain. Previous to putting the plaster-holder into the mouth, place a small wedge-shaped piece of plaster in a semi-plastic state, in the highest part of the roof of the mouth, its point of course touching first; as you press it up, it will gradually fill at every point around from the centre, without the possibility of there being any air-hole or imperfection thus far. This will generally fill the roof of the mouth down nearly on a level with the bottom of the alveolar ridge. Your holder, charged with comparatively a small quantity of plaster, is now introduced into the mouth; as it gradually presses upwards, it readily unites with the plaster already there, and flowing around the anterior surface of the gums, the union is complete at every quarter. On removing the plaster, you are always gratified to find the impression perfect in every respect.

"But it oftentimes, if not generally, occurs, especially if the above directions are followed in full, that great difficulty is experienced in removing the impression from the mouth, in consequence of atmospheric pressure. Sometimes we have been obliged to resort to the severest measures to separate the one from the other. No lifting one side or the other, no pressing backwards or forwards, would suffice. And when, after a good display of 'bone and sinew,' we have finally brought it away, tinctured with many a sanguineous stain, we have found a half-way apology for our severity in the comforting exclamation, that it was a '*remarkably* good impression.' But this is no late thing with us; we date it back to the *dark ages* of our practice; to our credit be it spoken, we soon discovered a remedy for this, which has ever since proven as infallible as it is satisfactory.

"In all of our holders for upper sets, we have a small hole punctured in them, about one line in diameter, at a point corresponding to the centre of the palatine arch. Just before filling one of these with plaster, we close up this orifice with a thin piece of sheet wax. Immediately on the plaster being pressed to its place in the mouth, we take a small piece of wire, and with it penetrating the wax, we pass it upward through the plaster, until it reaches the roof of the mouth; it is thus suffered to remain until the plaster is nearly or quite hard, when it is withdrawn. By this means, an air passage is formed to the centre of the impression, where the vacuum is always most complete, and the holder may be removed without the least difficulty.

"The crystallizing or hardening process of the plaster is wonderfully facilitated by using pure rain water, into which a small quantity of common salt or alum has been diluted. While the use of hard water simply, retards its setting, dissolving gum arabic or glue, in this last, will protract its setting to a very remarkable extent.

"We should avoid leaving our plaster in a damp place, or it may absorb moisture and soon become unfit for use; this can be dispelled, however, by heating it over again. Even plaster that has once been cast into forms, when pulverized and baked anew, is fit for use again.

"The imperfections occasioned by air-holes, in casting the plaster into moulds for the purpose of obtaining models, may be easily obviated by first placing a small quantity of thin batter into them, (we generally use a finer sort for this,) and then *blowing* down upon it a small current of air from the mouth, until we have displaced it, so that the surface of the mould, immediately under this stream of air, is nearly or quite discernible. We trace this little circlet, made by the force of the air from the mouth, displacing as we go, all over the surface of the mould. By this means, you drive every hidden bubble from its retreat, and at the same time force the plaster into the most delicate depressions of the mould. You then fill up the casting to the desired height, and on separating the model from the mould, you cannot but be delighted with the delicate perfection of your work.

"This method of blowing upon the plaster when in the form of a thin paste, is practised by sculptors and our best artists in plaster; by which means they obtain perfect impressions of the most delicate and elaborate works of art.

"The charming medallions and relievos in plaster, which occasionally come to our eyes, with their lines as sharp and delicate, and an exquisiteness of finish, as though they had come direct from the hand of the original artist, and were indeed archetypes themselves, were cast in this manner, for such perfection can be gained in no other way."

Impressions with Wax.—We have made repeated experiments with the different kinds of wax, to ascertain, if possible, the best for this purpose. The white wax, although the most cleanly in appearance, and the most inviting to be used about the mouth, yet, we have found it not to work so well as the yellow. We attribute this to the fact that it has lost a greater portion of its oil, by the process of bleaching; hence, is not so tenacious, and cracks more readily on the surface by cooling. We have, therefore, generally used the yellow bees-wax for taking impressions of the mouth, until, indeed, within the last one or two years, when we have occasionally substituted the adhesive wax, made according to the recipe given in No. 2, Vol. I, page 187 of the Dental Register. This article is more tenacious than either of the others and perhaps changes less after removal from the mouth.

In the use, however, of either, the first thing is to select the proper cup; this can be done, by trying one or more in the mouth. The second is, to get the wax the proper consistence. This should be sufficiently soft to adapt itself, by a gentle pressure, to all the irregularities of the gum. For impressions where temporary teeth are to be inserted, and the gums are yet soft and tender, it should be softer than where the mouth is ready to receive a permanent operation. In no case should the wax be so hard as to require a greater amount of force, to press it to its place, than is ordinarily used in the occlusion of the jaws, in mastication of our food. Pressure sufficient to flatten the ruga of the mouth, should never be used, as the impression will not be correct.

We have long noticed, and heard the remark from others, that those impressions which held to the mouth, by suction, most firmly, did not, as a general rule, give the best impressions. The force requisite for the disengagement of such, from the mouth, generally alters the form of the wax, and ofter partially draws it from the cup. To obviate this difficulty, we have adopted, for our wax impressions, the same plan recommended by Dr. Dwinelle, in the use of plaster, only making, where necessary, two or three punctures through the wax, instead of one through the plaster. After the wax has been thus disengaged, care should be taken to remove it from the mouth, so as not to disturb the wax around the border of the cup.

We have never found much benefit in taking cold water into the mouth, previous to the removal of the wax, nor in suddenly dipping it in cold water, immediately after its removal. We think the wax should be arranged in the cup, to suit the alveolar border, and the palital arch, so that when the pressure is first applied, the wax may press as equally as possible on the entire surface. An adherence to the suggestions, here recommended, we are sure will do much to remove the objections often made to the use of wax for this purpose.

Impressions in Gutta Percha.—In some few experiments, made with this article, we have found one or two objectiens, which induced us to lay it aside. In many mouths, the heat necessary to soften it, sufficient for getting a correct impression, without the application of too great force, is greater than can be well borne; and although we had a correct representation of the crowns of the teeth, as well as the necks, yet, we found that clasps fitted to them accurately, were too small for the teeth in the mouth, showing, as we thought, an evident contraction of the gutta percha by cooling, which, if it be the case, and to that extent apparent in these cases, would render it not so good an article for this purpose, as was at first supposed.—[Cin. Ed.

